# Food Safety Implementation and Associated Challenges: Insights from Cambodia’s Pangasius Fish and Chicken Farms

**DOI:** 10.3390/vetsci13040380

**Published:** 2026-04-15

**Authors:** Shwe Phue San, Linda Nicolaides, Sinh Dang-Xuan, Delia Grace, Stephen Young, Kuok Fidero, Chhoun Chamnan, Tumnoon Charaslertrangsi, Ra Thorng, Leab Kong, Rortana Chea

**Affiliations:** 1 Food and Markets Department, Natural Resources Institute, University of Greenwich, Medway, Chatham ME4 4TB, UK; ss6766m@greenwich.ac.uk (S.P.S.); l.nicolaides@greenwich.ac.uk (L.N.); d.c.randolph@greenwich.ac.uk (D.G.); stephen.young@greenwich.ac.uk (S.Y.); 2 International Livestock Research Institute, Hanoi 1000100, Vietnam; s.dang@cgiar.org; 3 National Meanchey University, Sisophon 10101, Cambodia; kuok.fidero@misti.gov.kh; 4 Fisheries Administration, No.186, Preah Norodom Blvd (42), Phnom Penh 12302, Cambodia; chhounchamnan@gmail.com; 5 Science Division, Mahidol University International College, Nakhon Pathom 73170, Thailand; tumnoon.cha@mahidol.ac.th; 6 CAPFish CAPTURE Project Office, Fisheries Administration, Phnom Penh 120101, Cambodia; rathorng@gmail.com (R.T.); kongleab@ymail.com (L.K.); 7 National Animal Health and Production Research Institute, General Directorate of Animal Health and Production, Phnom Penh 120603, Cambodia

**Keywords:** food safety practices, veterinary drug use, key informant interview (KII), mixed-methods approach, fishery and poultry farming, Cambodia

## Abstract

Since agriculture sustains most Cambodian households, food safety in the aquaculture and poultry sectors is a public health priority. This study investigates food safety practices and compliance with legal provisions through interviews with 20 participants across four provinces (Battambang, Siem Reap, Kampong Speu, and Takeo). The findings revealed critical deficiencies in hygiene, veterinary medical product management, and traceability. In addition to a detailed qualitative thematic analysis, Structural Equation Modelling identified veterinary drug control (β = 0.670, *p* < 0.001) and hygiene practices (β = 0.395, *p* < 0.001) as significant predictors of farm safety. While knowledge positively correlates with these practices, operational challenges negatively impact compliance (β = −0.466 and −0.497, *p* < 0.005). Notably, fish farmers reported using human medications (e.g., paracetamol) and often neglecting withdrawal periods due to limited VMP access and insufficient knowhow. While acknowledging the pilot-scale sample size, the findings of our modelling complement our qualitative analysis and provide a foundational model for future research. These findings suggest that farmer knowledge and systemic barriers are the primary drivers of food safety outcomes. The study highlights the urgent need for targeted training in good practices and responsible VMP use. Future research should explore scalable interventions to enhance regulatory compliance among smallholder farmers.

## Simple Summary

Ensuring safe food production in Cambodia’s fish and chicken farms is vital for public health, as most rural households depend on agriculture. This study investigates the challenges farmers face when trying to follow food safety rules. Researchers interviewed 20 participants across four provinces to understand how they manage their farms and comply with the Food Safety Law, 2022. The results show that while participants understand the importance of hygiene, they struggle with compliance costs and having limited time to participate in training. A major concern is the misuse of veterinary medicine; for example, some fish farmers use human drugs like paracetamol because they cannot find or read the labels on proper animal treatments. Furthermore, most farms do not keep records, making it impossible to track the movement of fish and poultry. To address these issues, the study recommends providing streamlined, participatory training on hygiene and the safe use of medicines. It also suggests improving access to animal health services and making rules easier for small farmers to follow. These changes will benefit society by reducing foodborne illnesses and improving the quality of food.

## 1. Introduction

Over 60 percent of households in Cambodia participate in agricultural activities, with crop cultivation, fishing, and poultry farming being the most common activities [1,2]. In Cambodian development policy discussions, aquaculture is increasingly recognized for its significant contribution to fulfilling fish demand and creating job opportunities in rural areas [3]. The freshwater fish farming sector is more advanced than marine aquaculture, with the most common species being *Pangasius* spp., followed by the giant snakehead (*Channa micropeltes*) and other species [4]. This focus on diversifying protein sources extends beyond aquaculture, as evidenced by the prominence of poultry farming, which primarily centres on chicken as the most widely raised species among agricultural households. In the 2019–2020 period, the National Institute of Statistics (NIS) reported populations of 40.8 million chickens and 25.1 million ducks [5].

Recent research highlighted gaps in hygiene and sanitation practices, as well as insufficient biosecurity measures, in the farming of fish and poultry [6,7]. Among those concerns, antibiotics are recognized as significant chemical contaminants that are abundantly deposited in the environment and, in turn, the food chain. It is well known that these antibiotics are widely used in both aquaculture and livestock farming. Moreover, while strict regulations in European countries may help mitigate the risks, the rising and often unregulated use of antibiotics in low- and middle-income countries (LMICs) remains a concern for food safety [8]. Furthermore, several studies have reported antimicrobial susceptibility, antimicrobial resistance (AMR), and the detection of extended-spectrum beta-lactamase (ESBL)-producing bacteria, including *Clostridium difficile*, *E. coli*, *Salmonella*, and *Campylobacter* species, in samples obtained from poultry, fish, and pork products [9,10,11,12,13,14,15].

The Royal Government of Cambodia enacted the Food Safety Law in 2022 [16] and established a comprehensive framework for monitoring and ensuring the safety, quality, hygiene, and compliance of food throughout the entire production chain. This framework aims to protect consumer health and encourage fair trade practices. While the law does not specifically focus on fish and poultry farming, it encompasses general regulations applicable to all food businesses, including those involved in farming. The provisions cover critical aspects such as hygiene standards, responsibilities of food business operators, traceability, and the authority of relevant agencies to perform inspections and enforce regulations, as well as strategies to prevent contamination [16]. One of the key food safety challenges in Cambodia is the discrepancy between safety regulations and their practical implementation at the farm level [17]. To improve food safety in informal high-risk food sectors such as perishable produce markets in Cambodia, stakeholders in the informal value chain must implement effective food safety practices [18]. This study aims to identify implementation gaps and systemic challenges within Cambodia’s poultry and fishery sectors by evaluating the demographic profile of farming businesses and assessing current food safety practices. Specifically, the research analyzed farmer compliance with the Food Safety Law, 2022, focusing on official control, hygiene protocols, and traceability systems while evaluating the responsible use of veterinary medicinal products and adherence to withdrawal periods. By synthesizing these elements, the study sought to provide an overview of the barriers to regulatory alignment and safety standards among smallholder farmers and fingerling suppliers in piloted provinces.

## 2. Methods

### 2.1. Study Design and Selection of Provinces

This study was designed as an exploratory effort to establish a foundational understanding of smallholder practices, serving as a pilot-scale framework to inform future, large-scale nationwide investigations. A desk review of the legal framework governing fish and poultry food control systems was conducted to develop and refine the technical guides for KIIs. Four provinces in Cambodia were selected as the study sites, with Siem Reap and Battambang designated for Pangasius farming due to their proximity to the Tonle Sap region and significant aquaculture potential. The Takeo and Kampong Speu provinces were selected for poultry farming, as their location within the plains region near the capital means they are strategic economic and industrial hubs. The selection of these locations was established through consultation with local experts and the supervisory team. Participant selection was based on farm accessibility and the voluntary willingness of farmers to engage in the research. To facilitate data collection, provincial officers from the Fisheries Administration (FiA) and the General Directorate of Animal Health and Production (GDAHP) coordinated with the farmers to arrange both semi-structured interviews and on-site observations.

In 2023, aquaculture production reached 24,880 metric tons (MT) in Siem Reap and 18,130 MT in Battambang. Concurrently, poultry production totaled 1,016,000 heads in Takeo and 5,630,120 heads in Kampong Speu (source: FiA and GDAHP, 2024).

The contents of the key informant interview (KII) were evaluated for user-friendliness through a pilot test involving two local individuals experienced in chicken and fish farming practices in Cambodia. The KII questions were formulated in accordance with the provisions of the Food Safety Law of Cambodia (2022) and the responsible use of antimicrobial compounds in the management of fish and chicken diseases, given increasing concerns about the detection of antimicrobial-resistant bacteria in POAO in Cambodia.

**Figure 1:**
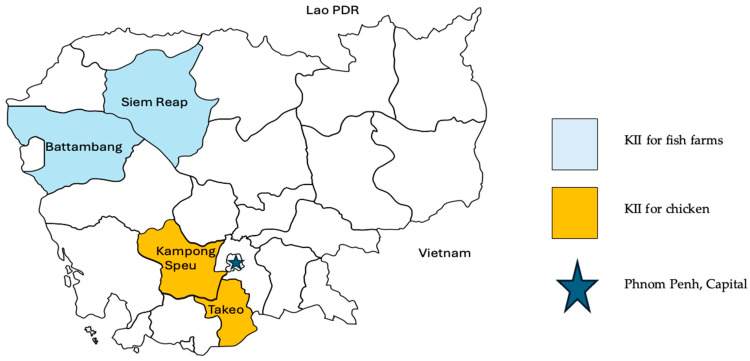
Locations of 4 provinces from which farmers were selected in Cambodia.

Participants aged 18 or older were briefed on the purpose of the KII and were asked to provide written informed consent if they agreed to participate. Participants were also informed that their answers would remain anonymous, that they could withdraw at any time, and that the information collected would be kept confidential. The interview was conducted in the Khmer language and translated into English for validation and confirmation by the subject matter experts. The KIIs were conducted in July 2024 in Siem Reap and Battambang for fish farming, followed by site visits to the Takeo and Kampong Speu provinces in August 2024 for chicken farming, as illustrated in Figure 1.

### 2.2. Study Population

A list of fish farmers, including the areas of the fishponds and production volumes, was acquired from the FiA [19]. The study engaged five participants from each sector per province; for poultry, this included five chicken farmers, while the aquaculture participants comprised four grow-out farmers and one juvenile fish (fingerling) supplier. With the support of the provincial FiA officers, the selected farmers in Siem Reap and Battambang were contacted and KIIs were arranged. Similarly, five chicken farmers in Kampong Speu and five in Takeo province were chosen for interview based on recommendations from local experts and provincial officers for animal health and production (POAHP), with a view to representing different farm scales.

### 2.3. Conducting Key Informant Interviews (KII)

In July 2024, interviews were conducted with four fish farmers and a fingerling supplier in Siem Reap, and with another four fish farmers and a fingerlings supplier in Battambang province. The interviews were performed face-to-face, which was convenient for the participants. Written consent forms from participants were obtained prior to the interviews, photographs, and audio recordings. Similarly, in August 2024, five chicken farmers in Takeo province and five in Kampong Speu province were interviewed. The participants included commercial producers (2 × fish farms and 2 × chicken farms)and farmers operating as small-scale and backyard producers (*n* = 16). The interviews were recorded to clarify participants’ answers and were deleted after use. During the interview process, the pre-formulated questions (Appendix A) were accompanied by probing questions. The findings and responses were translated from Khmer to English and later polished without altering the original meanings.

### 2.4. Quality Control and Data Analysis

To ensure the rigour and credibility of the qualitative analysis, a robust inter-coder reliability (ICR) framework was implemented throughout the data processing phase. Primary data collection was conducted by two local experts specializing in fisheries and poultry safety, who subsequently collaborated on the joint preparation of the interview transcripts. To maintain high interpretive consistency, more experienced researchers within the fishery and poultry sectors performed comprehensive quality checks. Furthermore, as a final component of the quality control protocol, the overall accuracy and thematic integrity of the transcripts were reviewed by the supervisory team to minimize researcher bias and verify the reliability of the findings.

The data collected in Khmer were translated into English by the local experts and verified by a local research supervisory team member. The demographic details and open-ended responses from the English transcript were input into the relevant rows of an Excel table. The study employed a mixed-methods analytical approach to ensure a comprehensive interpretation of the data. While the primary analysis was qualitative, descriptive quantitative measures were integrated to provide a structural baseline for the findings. Despite the focused sample size (*n* = 20), this dual approach was utilized to triangulate the data to explore the complex socio-technical dynamics of the Cambodian fishery and poultry sectors.

The data were organized into six main thematic categories, each representing a key aspect of farm management and food safety implementation. The six categories and respective coding for the data analysis are shown in Table 1.

To explore the structural relationships between variables, SEM was performed using the Maximum Likelihood (ML) estimator. Given the pilot-scale nature of the research (*n* = 20) and the ordinal characteristics of the data, the model was constructed using a robust Spearman non-parametric correlation matrix as the primary input rather than raw data. This approach was selected to account for non-normal distributions and to provide a more stable foundation for the model fitting process than parametric correlations would afford. The dataset used for quantitative analysis is attached as Appendix B.

Cronbach’s alpha was also used to assess the internal consistency reliability of the ten-item scale measuring farmers’ food safety knowledge and practices. The ten items included in Cronbach’s alpha analysis were “Type of farmers (Female = 0 and Male = 1 and for the rest: Yes = 1, No = 0)”, “Participation in food safety training”, “Following withdrawal period”, “Record keeping practice”, “Traceability implementation”, “Knowledge of GHP benefits”, “Farm hygiene implementation”. “Implementation of basic storage hygiene”, “Basic worker health”, and “Basic knowledge of allergen”.

## 3. Results

### 3.1. Demographic of the Studied Participants

Table 2 presents the comparison of key demographic and operational characteristics between fish farmers and fingerling suppliers (*n* = 10) and chicken farmers (*n* = 10) in Cambodia, offering insights into the sex, age, education levels, farm sizes, and household dynamics of the chicken and fish farming sectors.

The data shown in Table 2 reveals differences in gender distribution, age group, education, farm scale, and family size within the scope of our pilot study, which may influence farming practices, productivity, and economic outcomes.

#### 3.1.1. Gender Distribution and Workforce Composition

The findings revealed the gender disparity between the two farming sectors (See Table 2). All surveyed fish farmers (100%) within our study were male, indicating the possibility that aquaculture in this region remains a male-dominated industry. In contrast, chicken farming showed a balanced gender distribution, with 50% male and 50% female participation. This suggests that poultry farming may be more accessible or culturally acceptable for women in Cambodia, possibly because of lower physical labour requirements or smaller operational scales. Overall, the combined data shows that 75% of the surveyed farmers were male. While 75% of the total participants were male, this observation reflects the specific demographics of our study cohort and should be interpreted with caution, as a larger sample size would be required to confirm a nationwide trend of male predominance.

#### 3.1.2. Age Structure and Generational Involvement

In our study, the majority of farmers in both groups fell within the 30–60 age group (70%), indicating that middle-aged individuals dominate both fish and chicken farming (See Table 2). However, in fish farming, a slightly higher proportion of farmers were elderly (30% over 60 years old) than in chicken farming (20%). Only one young farmer (under 30) was engaged in chicken farming, while none were recorded in fish farming. This suggests a potential lack of youth engagement in both sectors, which could pose challenges for future sustainability and innovation. Encouraging younger generations to enter agriculture through training programmes and financial incentives may be necessary to ensure long-term sector growth. Nevertheless, as this is a pilot-scale cross-sectional study with a limited sample size, these age-related observations should be considered preliminary and would require validation through a larger, representative and longitudinal survey to confirm broader demographic trends.

#### 3.1.3. Education Levels and Knowledge Base

Education plays a crucial role, enhancing farming efficiency and promoting the adoption of technology (see Table 2). The data shows that fish farmers generally had lower education levels, with 60% having only primary education and just 10% attaining tertiary education. In contrast, 40% of chicken farmers had a tertiary education, suggesting a more educated workforce in poultry farming. This difference may influence farming techniques, disease management, and business practices, with chicken farmers potentially adopting more modern and efficient methods. Overall, 45% of all farmers had only primary education, suggesting that basic training and literacy programmes could improve productivity across both sectors.

#### 3.1.4. Family Size, Farm Size and Operational Scale

The mean family size was 5.5 (median = 5.0) for fish farmers and 4.8 (median = 5.0) for chicken farmers (Table 2). Regarding operational scale, the cohort consisted primarily of small-scale chicken farms (60% < 1 ha) and medium-sized fish farms (50% 1–10 ha). As these proportions reflect a sampling design intentionally chosen to capture a range of farm sizes, these data describe the specific study participants rather than a systematic sectoral comparison. Consequently, these findings provide context for the operational challenges faced by these specific small- and medium-scale holders rather than representing broader national trends in land requirements.

### 3.2. Findings from Structural Equation Modelling (SEM)

**Figure 2:**
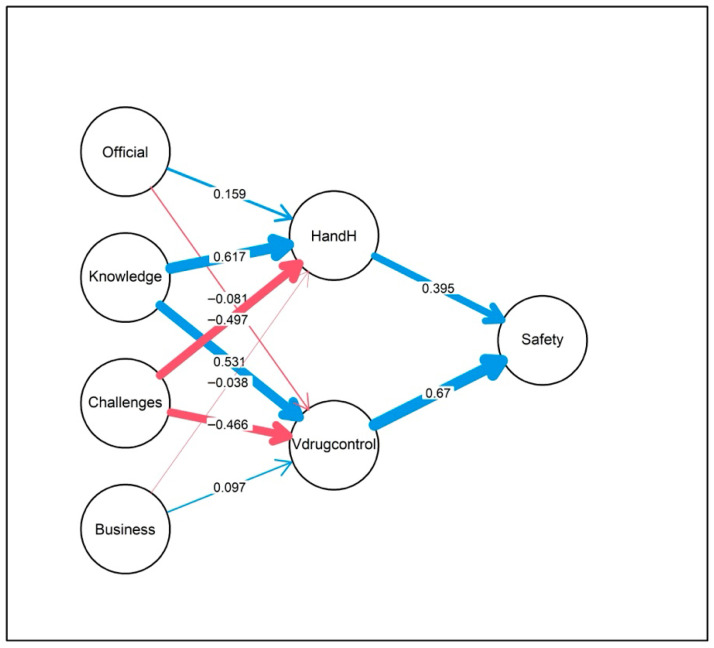
Structural Equation Modelling for the safety of fish and chicken at farm level, where Official = official control, Knowledge = basic food safety and hygiene knowledge, Challenges = food safety implementation challenges, Business = fish/chicken farming business, HandH = health and hygiene, Vdrugcontrol = veterinary drug control, and Safety = food (fish/chicken) safety.

As shown in Figure 2, the analysis used a SEM approach with the “lavaan package” in R Studio R4.4.1 [20]. In this model, product safety is regressed on two key operational practices, which are veterinary drug control and health and hygiene. In turn, both mediating constructs are predicted by four antecedents: business aspects, food safety implementation challenges, knowledge, and official control. This multistep approach allows us to understand both the direct effects on product safety and the indirect pathways through which underlying factors operate. A detailed exposition of the goodness-of-fit criteria employed for the Structural Equation Model is provided in Appendix C.

The key points of Figure 2 are the direct impact on veterinary drug control and health/hygiene practices directly and positively affecting product safety. Indirect impact was observed as knowledge boosts both operational practices, while challenges impede them. Since these practices in turn influence product safety, knowledge and challenges have important indirect effects on the outcome. The non-significance of business and official control in predicting the mediators suggests that improvements in product safety should be prioritized through interventions focused on overcoming challenges and building knowledge, rather than solely relying on business environment modifications or official regulatory controls.

**Figure 3:**
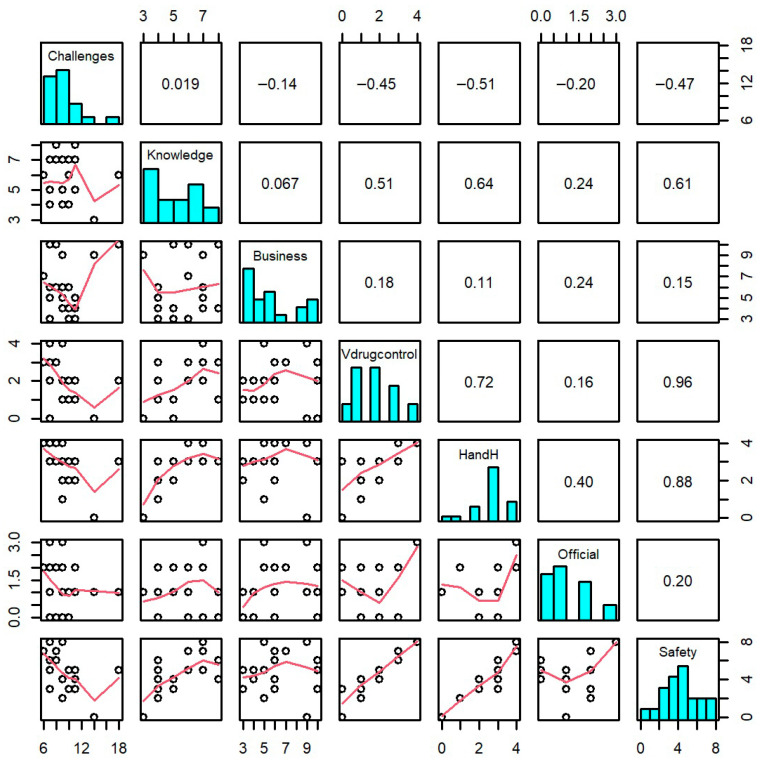
Relationship matrix of farm safety variables. The bar charts (**diagonal**) show the distribution of data for each category. The numbers (**top right**) indicate how strongly two factors are related, while the dots and lines (**bottom left**) provide a visual map of those relationships.

As shown in Table 3, both predictors are highly significant (*p* < 0.001), demonstrating that better veterinary drug control and improved hygiene both enhance product safety. However, veterinary drug control has a stronger impact (0.670) than hygiene (0.395), suggesting it is the more effective priority for safety interventions. Additionally, farmer knowledge significantly improves drug practice (0.531, *p* = 0.001), while operational challenges significantly hinder it (−0.466, *p* = 0.005). The correlation matrix of food safety variables is presented in Figure 3.

Cronbach’s alpha of 0.7964 in our study indicates good reliability, indicating that the scale produces consistent results. By confirming strong inter-item correlations, the analysis supports the scale’s validity for research and assessment purposes.

### 3.3. Findings from Qualitative Analysis

The qualitative analysis revealed the level of food safety practices among farmers, focusing on hygiene, veterinary drug use, and traceability based on the Food Safety Law (2022).

#### 3.3.1. Awareness of the Benefits of Good Hygiene Practices (GHPs)

Fish farmers recognize that GHPs help maintain clean water, reduce bad smells, and reduce the risk of bacterial or viral contamination to some extent, helping prevent fish diseases and promoting clean, healthy conditions for fish. They also note that they protect consumers from illness and supports environmental sustainability.

Chicken farmers highlight that GHPs reduce disease risks, improve poultry health, and increase profits. They emphasize that good hygiene results in safer products, prevents the spread of disease, and ensures healthier conditions for both workers and livestock. Overall, farmers in both sectors agree that GHPs enhance productivity, product quality, and sustainability while protecting public health. A small-scale chicken farmer in Takeo province noted,

“Following GHP ensures high-quality products and prevents diseases.”

#### 3.3.2. Implementation of Hygiene Practices

Both fish and chicken farmers reported challenges in maintaining hygiene standards at farms due to limited resources, such as time constraints, lack of knowledge, and difficulties in training workers. For instance, a medium-scale chicken farmer in Kampong Speu province noted,

“We clean the drinkers twice a day and the feeder once per cycle, but workers sometimes fail to comply with hygiene rules such as hand washing and cleaning the utensils and equipment.”

This highlights the need for targeted training programmes to improve compliance. The majority of fish farmers said they do not have a regular cleaning schedule at their farms; however, a small-scale fish farmer in Siem Reap province described the pond cleaning process in a comparable manner, stating,

“Once the fish are harvested, the pond’s water is drained. Lime is used to disinfect the pond, which is then allowed to dry for two weeks. After this period, water is reintroduced into the pond, and a few days are permitted to elapse before stocking the fingerlings. After a growth period of eight to ten months, the fish are ready for harvest and distribution to local markets.”

Lime is frequently utilized in both fish and poultry farming as it is considered as an effective sanitization tool.

#### 3.3.3. Veterinary Drug Use and Control

According to the responses from fish and chicken farmers, improper use of veterinary drugs was widespread. A few fish farmers reported using human medicines such as paracetamol and amoxicillin mainly because of limited knowledge, adherence to traditional ways of thinking, and inadequate veterinary services. A small-scale fish farmer in Battambang province stated

“We buy veterinary drugs imported from neighbouring countries, but we cannot read the labels, so we don’t follow withdrawal periods.”

Another small-scale fish farmer in Siem Reap province said

“I use more than 10 different types of medicines, such as growth promoters, treatments for diseases, water quality products, and feeds. The sellers often change the brands or the packaging of the medicines.”

A medium-scale fish farmer in Battambang province explained the common practice at his farm:

“For fish growth, I usually combine 3 medications into the feed once a week for a period of approximately 1–3 months. For fish diseases, such as swollen eyes and stomach, I administer amoxicillin, ampicillin, and paracetamol. These medications are in powdered form, and I utilize 2 spoons for every 10 kg of feed. I provide this for about 5 days, depending on the fish’s condition.”

The trend of veterinary drug use is also similar in the chicken sector, as one small-scale chicken farmer in Kamping Speu province noted:

“I don’t know the names of the drugs or chemicals I am using at the farm. All of them have no label or description on the bottle. I bought the medicine from the veterinary shop. I follow the seller’s instructions.”

However, chicken farmers in Takeo province are committed to the responsible use of veterinary drugs, including proper cold storage facilities and appropriate record-keeping. This underscores the risks of antimicrobial resistance (AMR) and the need for improved access to veterinary services, including accurate prescriptions, the use of approved veterinary medicines, and clear labelling in local languages.

#### 3.3.4. Traceability and Record-Keeping

Traceability systems have not been widely implemented, particularly in fish farming. Only one chicken farmer in Kampong Speu and another in Takeo province supply their products to well-known companies that keep records of farming practices, such as veterinary drug usage, harvest/slaughter dates, input purchases, and sales. A fish farmer noted that the traceability system (one step back and one step forward) is not commonly practiced, with some stating they lack the time to implement it. Additionally, a small-scale chicken farmer in Takeo province explained,

“We know our input suppliers and customers, but don’t maintain any records or documents regarding suppliers or buyers.”

This absence of a traceability system implementation hampers efforts to monitor food safety and address contamination incidents and does not comply with the requirements of Article 12 of the Food Safety Law (2022). Food business operators are obliged to immediately notify and collaborate with competent authorities to mitigate risks through the implementation of robust traceability, food recall, and withdrawal procedures.

#### 3.3.5. Official Control and Enforcement

The interviews revealed mixed experiences with inspections among fish and chicken farmers. Some reported recent and regular inspections, while others indicated no or infrequent visits. The dates of the last inspections ranged widely (2017–February 2024), suggesting some farms receive consistent monitoring, while others face long periods without official engagement, possibly due to resource limits or targeted approaches. A notable inconsistency was how inspection findings were discussed. Uninspected farmers generally had limited knowledge of their neighbours’ experiences, though one aware farmer reported that inspections were common among small fish farmers. The analysis of the answers reveals an operational but uneven inspection system in execution, communication, and coverage, indicating crucial areas for policy intervention to boost food safety effectiveness. The study identified several challenges hindering the food safety implementation practices of fish and chicken farmers as follows.

##### Business Expansion Plan and Barriers

Some farmers mentioned substantial barriers, such as financial limitations, including a lack of capital, that are frequently cited as prohibitive factors. Spatial constraints, such as insufficient land or limited farm space, further restrict growth opportunities. External risks, particularly weather-induced fish diseases, also deter expansion efforts. Additionally, rising feed prices undermine profitability despite growing market demand. Conversely, those planning to expand often cite economic incentives, such as higher profits, alongside social objectives, such as knowledge-sharing among farmers. Both fish and chicken farmers face several obstacles in sustaining their operations, with market access and price fluctuations emerging as primary concerns. Financial burdens, such as high sanitization costs, add additional strain, while poultry farmers specifically struggle with odour control and maintain livestock health.

- Record-Keeping Practices

The adoption of systematic record-keeping remains low among farmers, primarily due to time constraints, lack of awareness, and language barriers. Many respondents consider documentation an uncommon practice, particularly when labelling for aquatic medicines is not in their native language. A small subset acknowledges the value of record-keeping but struggles with implementation amid demanding workloads.

- Complaint and Recall Systems

Farmers typically transfer responsibility to buyers once products are sold, relying on them to inspect and assume liability for any issues. Documented recall procedures are rare, with most farmers believing such measures are impractical for perishable goods, which are often consumed before problems are identified. Complaints related to food safety are uncommon, with reported issues more frequently concerning product weight or non-safety-related defects. Some poultry farmers depend on corporate partners, such as the contractor, to conduct lab tests, further distancing them from direct accountability.

- Inspection and Regulatory Challenges

Both the chicken and fish sectors exhibit inconsistencies in regulatory oversight, with limited direct engagement from authorities. Farmers frequently operate without systematic guidance, relying instead on informal networks for critical decisions. In aquaculture, for example, health issues such as cataracts, skin lesions, and fin deterioration are often addressed through trial-and-error treatments, including the use of human medications, due to insufficient access to veterinary expertise. Similarly, poultry farmers confront challenges like respiratory infections and leg disorders, which are frequently managed through ad hoc remedies rather than standardized protocols. Transparency remains a concern, as farmers rarely receive feedback on product testing, whether for fish or poultry. Many farmers depend on peer advice for medication use, particularly when labels are in foreign languages, increasing the risk of improper dosages or unintended residues. Compliance with formal guidelines such as the Good Aquaculture Practices (GAqPs) in fish farming or biosecurity measures in poultry production is inconsistent [21]. Some farms follow basic protocols, while others prioritize immediate problem-solving over preventive measures.

- Key Themes of the Challenges and Implications

Several recurring themes emerge from these findings. Structural barriers, including financial constraints and regulatory gaps, impede both operational efficiency and food safety compliance. Informal practices dominate labour, disease management, and administrative tasks, reflecting a reliance on ad hoc solutions rather than standardized protocols. Market instability, driven by price volatility and import competition, discourages long-term investments in quality improvements. The use of imported veterinary drugs with labels in foreign languages posed a significant barrier. Farmers could not follow withdrawal periods or dosage instructions, increasing the risk of drug residues in food products. A medium-scale fish farmer in Siem Reap province stated,

*“Input prices are rising, and we need government support to access affordable inputs.”*

Addressing these economic barriers is crucial for sustainable improvements in food safety. Knowledge gaps persist regarding residue testing, recall procedures, and proper use of medication. Finally, the prevailing assumption that buyers bear post-sale risks reduces farmers’ incentives to adopt more rigorous safety measures. Addressing these challenges will require targeted interventions in financing, education, and policy enforcement to enhance sustainability and food safety in small-scale farming.

## 4. Discussion

This study integrates SEM, Cronbach’s alpha, and thematic analysis to highlight key strengths, barriers, and policy implications. Demographically, aquaculture is predominantly male dominated, whereas chicken farming shows gender balance and a more educated workforce with frequent tertiary qualifications. While a regional study reported that poultry farmers often possess adequate safety knowledge despite persistent hygiene misconceptions, that study found no statistically significant demographic differences regarding gender or age [22].

Farmers articulated that GHP contributes to maintaining hygienic environments, mitigating disease prevalence, enhancing product quality, safeguarding consumer health, and potentially improving market prices. The observation that workers sometimes fail to comply with the basic hygiene guidelines of Codex [23] indicates gaps that should be filled by more practical, experiential training and potentially simpler, more readily adoptable protocols for behavioural change. The traditional pond-cleaning process described by fish farmers, involving lime disinfection and drying, reflects established practices but may not consistently align with comprehensive modern GHP standards without additional measures. A systematic review [24] indicated that a critical challenge for improving farm hygiene in low- and middle-income countries (LMICs) is the scarcity of systematically gathered evidence on effective hygiene management strategies, despite the significant role of agricultural communities globally.

While some corporate chicken farmers maintain proper storage and records, most producers face significant knowledge deficits and limited veterinary access. The most critical concern is the misuse of human medications, like paracetamol and amoxicillin. Reliance on vendor advice and incomprehensible imported labels leads to neglected withdrawal periods, creating severe risks for drug residues and antimicrobial resistance. Inadequate legal enforcement and limited GAqP awareness further jeopardize food safety and industry sustainability [25].

This informal approach to use of veterinary medicines directly disregards the principles of responsible use and the objectives stipulated in the Food Safety Law. The SEM analysis robustly supports this observation, demonstrating that “Knowledge” exerts a significant positive effect on “Vet drug control,” while “Challenges” exhibit a significant negative effect. The lack of statistical significance regarding business and official controls suggests that improving veterinary drug safety depends more critically on addressing knowledge gaps and operational challenges among producers. The easy availability of antibiotics, along with the barriers to accessing quality veterinary care and preventive measures, likely leads to the inappropriate use of antibiotics in complex manners [26,27,28]. In rural provinces, a significant number of residents reported a willingness to share human antibiotics with household animals or livestock, with the highest prevalence (59.3%) observed in Prey Veng [29]. In Cambodia, limited access to professional guidance in pig farming often leads to unintentional antimicrobial misuse, creating significant challenges for managing resistance. When waste disposal and withdrawal periods are not fully addressed, resistant bacteria can spread through the local environment [30]. This affects neighbouring fish and chicken sectors, as shared water and soil allow resistance to reach different species. Improving regional food safety will likely require collaborative support and shared monitoring across all livestock industries to protect communal health [31].

The extensive misuse of veterinary antibiotics has been widely documented in numerous studies around the world. Notably, research from Kenya and China indicates that substantial antibiotic deployment in livestock farming and aquaculture has precipitated widespread misuse, resulting in concerning levels of antibiotic residues and considerable environmental pollution. In response, the implementation of comprehensive regulatory measures is advocated. These include rigorous enforcement of existing legislation, fostering the development of antibiotic alternatives, and integrating advanced supervisory technologies to encourage responsible antibiotic stewardship and thereby safeguard public health [32,33,34].

While Cambodia’s 2022 Food Safety Law provides a helpful framework, practical challenges at the farm level make full compliance difficult. Many farmers face constraints in registration and traceability due to limited time or language barriers, and independent producers often lack the same structural incentives as commercial ones. Currently, regulatory support remains inconsistent, and the absence of clear guidance often leads farmers to seek informal advice. However, the present results suggest that providing direct educational support and practical resources may be more effective than official controls alone in helping farmers overcome financial barriers and improve long-term sector sustainability.

Our findings indicate limited engagement among young farmers (<30 years) in the fishery and poultry sectors, possibly due to low perceived profitability and the labour-intensive nature of traditional agriculture. The lack of modern technological integration may further diminish the sector’s appeal to the youth. To ensure development sustainability, future research should investigate Cambodian youth aspirations and the agri-food sector’s potential. Policies should prioritize transitioning from survivalist farming to technology-driven agribusiness to better engage this demographic.

## 5. Conclusions

This pilot study provides preliminary insights into food safety challenges within specific chicken and Pangasius farming cohorts in Cambodia. While the results indicate a foundational awareness of GAqP and Good Animal Husbandry Practices (GAHPs) among these farmers, practical implementation appears constrained by structural barriers, informal practices, and localized knowledge deficits. The SEM analysis suggests that enhancing farmer knowledge and addressing operational hurdles could be effective pathways for improving veterinary drug management and hygiene practices.

To support the development of these sectors, the findings suggest the potential benefit of integrated strategies, such as practical training delivered in local languages and the expansion of community-based veterinary services. Addressing linguistic barriers through improved enforcement on labelling or the use of pictograms could further support compliant drug utilization. Additionally, exploring financial mechanisms like micro-loans may help smallholders invest in the infrastructure necessary for hygiene upgrades. Rather than strictly punitive measures, a shift toward a supportive regulatory environment focusing on risk-based control may better encourage farmer participation in safety standards.

The introduction of simplified traceability systems and enhanced coordination between relevant ministries, such as the Ministry of Agriculture, Forestry, and Fisheries (MAFF), Ministry of Health (MOH), and Ministry of Commerce (MOC), could strengthen the “farm-to-fork” safety network. While these observations are based on a limited sample, they highlight important areas for further large-scale research. By aligning future interventions with the practical realities of small-scale farmers, there is an opportunity to foster a more resilient and sustainable food safety environment in the region.

Finally, to operationalize these findings, the Royal Government of Cambodia could consider institutionalizing secondary legislation under the Law on Animal Health and Production and the Fisheries Law. Such a framework, supported by international partners like the United Nations Industrial Development Organization (UNIDO), Food and Agricultural Organization of the United Nations (FAO), German Agency for International Cooperation (GIZ), and WorldFish, would benefit from national budget allocations to strengthen human resources and quality infrastructure. Success may be measured by metrics such as farm registration rates and the frequency of risk-based inspections. Ultimately, while this pilot study provides foundational evidence for improving the safety and sustainability of the chicken and Pangasius sectors, future nationwide research is needed to validate these interventions, evaluate compliance costs, and assess the role of consumer demand in driving safer farming practices.

## Figures and Tables

**Table 1:** Thematic grouping with respective variables and coding for Structural Equation Modelling (SEM).

Thematic Category	Variable	Coding and Measurement
Socio- Demographics (Potential Risk Factors)	Gender	Female = 0; Male = 1
	Age	<40 years = 1; 40–50 years = 2; >50 years = 3
	Family Size	<3 Members = 1; 3–7 Members = 2; >7 Members = 3
	Farm Proximity	On-Farm = 1; <5 km = 2; >5 km = 3
Knowledge	Experience	<10 years = 1; 10–20 years = 2; >20 years = 3
	Education Level	No Formal = 0; Primary = 1; High School = 2; University = 3
	Allergen Knowledge	Yes = 1; No = 0
	Hygiene & Biosecurity Knowledge	Yes = 1; No = 0
Farm Business Profile	Farm Size	Small = 1; Medium = 2; Large = 3
	Production Volume	<10 MT (or 1000 Heads) = 1; >10 MT = 2
	Number of Workers	<5 = 1; 5–10 = 2; >10 = 3
	Expansion Plans	Yes = 1; No = 0
Veterinary Drug Control	Direct drug Use	Yes = 1, No = 0
	Follow Withdrawal Period	Yes = 1, No = 0
	Traceability System	Yes = 1, No = 0
	Recording Keeping	Yes = 1, No = 0
Health & Hygiene	Farm Hygiene	Yes = 1, No = 0
	Storage Hygiene	Yes = 1, No = 0
	Worker Health and Hygiene	Yes = 1, No = 0
	Traceability System	Yes = 1, No = 0
	Record-Keeping	Yes = 1, No = 0
Official Control	Farm Registration	Yes = 1, No = 0
	Inspection by Competent Authority	Yes = 1, No = 0

**Table 2:** Demographic overview of the selected fish and chicken farmers.

Variable	Category	Fish Farmers (*n* = 10)	Chicken Farmers (*n* = 10)	Total (*n* = 20)
Sex	Male	10 (100%)	5 (50%)	15 (75%)
	Female	0 (0%)	5 (50%)	5 (25%)
Age Group	Under 30	0 (0%)	1 (10%)	1 (5%)
	30–60	7 (70%)	7 (70%)	14 (70%)
	Over 60	3 (30%)	2 (20%)	5 (25%)
Education	No formal education	1 (10%)	0 (0%)	1 (5%)
	Primary school	6 (60%)	3 (30%)	9 (45%)
	High school	2 (20%)	3 (30%)	5 (25%)
	College/university	1 (10%)	4 (40%)	5 (25%)
Farm Size	Small (<1 ha)	4 (40%)	6 (60%)	10 (50%)
	Medium (1–10 ha)	5 (50%)	3 (30%)	8 (40%)
	Large (>10 ha)	1 (10%)	1 (10%)	2 (10%)
Family Size	Mean (range)	5.7 (4–10)	4.4 (3–6)	5.1 (3–10)

**Table 3:** Estimates and probabilities values of each antecedent toward product safety.

	Estimate	Std. Err	z-Value	*p*-Value
**Product safety**				
Vet drug control	0.67	0.03	22.347	<0.0001
Health and hygiene	0.395	0.03	13.165	<0.0001
**Vet drug control**				
Business	0.097	0.166	0.584	0.559
Challenges	−0.466	0.165	−2.822	0.005
Knowledge	0.531	0.166	3.206	0.001
Official control	−0.081	0.173	−0.469	0.639
**Health and hygiene**				
Business	−0.038	0.125	−0.305	0.761
Challenges	−0.497	0.124	−4.015	<0.0001
Knowledge	0.617	0.124	4.962	<0.0001
Official control	0.159	0.13	1.228	0.219

## Data Availability

The original contributions presented in this study are included in the article/Supplementary Materials. Further inquiries can be directed to the corresponding author.

## Supplementary Materials

The following supporting information can be downloaded at: https://www.mdpi.com/article/10.3390/vetsci13040380/s1.

## Abbreviations

The following abbreviations are used in this manuscript:

AMR	Antimicrobial Resistance
ASEAN	Association of Southeast Asian Nations
BTB	Battambang Province
CA	Competent Authority
CAC	Codex Alimentarius Commission
CAST	Commercialization of Aquaculture for Sustainable Trade
CF	Chicken Farmer
CQS	Cambodia Quality Seal
DAD	Department of Aquaculture Development, Fisheries Administration
DG	Director General
ESBL	Extended-Spectrum Beta-Lactamase
EU	European Union
FCA	Fishery Competent Authority
FES	Faculty of Engineering and Science
FF	Fish Farmer
FiA	Fisheries Administration
FREC	Faculty Research Ethics Committee
GAP	Good Agricultural Practice
GAqP	Good Aquaculture Practice
GAHP	Good Animal Husbandry Practice
GDAHP	General Directorate of Animal Health and Production
GHP	Good Hygiene Practice
KII	Key Informant Interview
KPS	Kampong Speu Province
MAFF	Ministry of Agriculture, Forestry and Fisheries
NFCS	National Food Control System
NIS	National Institute of Statistics
NRI	Natural Resources Institute
NRMP	National Residue Monitoring Plan
SEM	Structural Equation Modelling
SR	Siem Reap Province
TK	Takeo Province
UNIDO	United Nations Industrial Development Organization
UOG	University of Greenwich
USDA	United States Department of Agriculture

## Appendix A. KII Questionnaire

**BASIC INFORMATION**	
Date of interview |____|____|/|____|____|/2024 (Day/Month/Year)	
Unique ID of farmer	
Sex	Female
	Male
	Not identified
Age	
Education level	Primary
	Secondary (High school)
	Tertiary (College/University)
Family size	
Address	
Walking distance between home and the farm (km)	
Walking time between home and the farm (minutes)	
What is the source of main income?	
Toal farm size (ha)	Total land size: ………………..(ha) Owned Land: ……………………(ha) Rented land: …………………….(ha)
Species/varieties of fish/chicken	
Production volume (kg) per month	Species 1: …………………kg Species 2: …………………kg Species 3: …………………kg
Years of experience in farming	
Type of farming	Fish/Chicken only
	Integrated farming
No. of workers	Full Time
	Casual
Target market	Domestic
	International
Do you have any plan to expend your farming business?	
Please explain why yes or no.	

## Appendix B. Dataset Used for SEM

**Type**	**Basic Worker Health**	**Sex (M = 1, F = 0)**	**Family Size**	**Disgp**	**Agegp**	**Challenges**
CF5	1	1	3	1	1	6
CF9	1	1	5	1	1	8
CF2	1	1	4	1	1	7
CF10	1	1	3	3	2	9
FF8	1	1	2	3	1	7
CF7	1	0	4	1	2	7
CF3	1	0	5	1	3	9
FF5	1	1	4	2	3	10
CF6	1	1	4	1	3	9
FF3	0	1	8	3	2	14
FF4	1	1	6	2	2	11
CF8	1	0	5	1	1	7
FF2	1	1	5	2	1	9
CF4	1	0	6	1	3	10
FF1	1	1	7	2	1	11
CF1	1	0	5	1	2	8
FF7	1	1	10	4	3	18
FF9	1	1	6	1	3	11
FF6	1	1	4	2	2	9
FF10	1	1	5	1	3	10
Type	expgp	Food safety training	Education	Basic knowledge of allergen	Knowledge of GHP benefits	Knowledge
CF5	1	1	3	0	1	6
CF9	1	1	3	1	1	7
CF2	1	0	2	0	1	4
CF10	1	1	3	1	1	7
FF8	1	1	1	1	1	5
CF7	1	1	1	0	1	4
CF3	1	0	2	0	1	4
FF5	1	1	1	0	1	4
CF6	2	1	1	0	1	5
FF3	2	0	1	0	0	3
FF4	2	0	1	1	1	5
CF8	2	1	3	0	1	7
FF2	2	1	1	0	0	4
CF4	3	1	1	0	1	6
FF1	3	1	2	0	1	7
CF1	3	1	2	1	1	8
FF7	3	1	1	0	1	6
FF9	3	1	2	1	1	8
FF6	3	0	0	0	1	4
FF10	3	1	1	1	1	7
Type	farmsizegp	prodgp	workergp	Plan to expand the business	Business	
CF5	3	3	1	0	7	
CF9	2	2	1	1	6	
CF2	2	2	1	1	6	
CF10	3	3	2	1	9	
FF8	3	3	3	1	10	
CF7	1	1	1	0	3	
CF3	2	1	1	0	4	
FF5	1	2	3	0	6	
CF6	1	1	1	1	4	
FF3	3	3	2	1	9	
FF4	1	1	1	0	3	
CF8	1	2	1	1	5	
FF2	2	2	1	0	5	
CF4	1	1	1	0	3	
FF1	2	2	1	0	5	
CF1	3	3	3	1	10	
FF7	3	3	3	1	10	
FF9	1	2	1	0	4	
FF6	2	2	1	1	6	
FF10	1	2	1	0	4	
Type	Use of antibiotics	Withdrawal period	Traceability	Record-keeping	Vet drug control	
CF5	1	1	0	1	3	
CF9	1	1	0	1	3	
CF2	1	1	1	0	3	
CF10	1	1	1	1	4	
FF8	0	0	0	0	0	
CF7	1	1	0	0	2	
CF3	1	0	0	0	1	
FF5	1	0	0	0	1	
CF6	1	0	0	0	1	
FF3	0	0	0	0	0	
FF4	1	0	0	0	1	
CF8	1	1	1	1	4	
FF2	1	0	0	0	1	
CF4	1	1	0	0	2	
FF1	1	1	0	0	2	
CF1	1	1	1	0	3	
FF7	1	1	0	0	2	
FF9	1	0	0	0	1	
FF6	1	1	0	0	2	
FF10	1	1	0	0	2	
Type	Farm hygiene	Basic storage hygiene	Basic worker health	Record-keeping	Health and hygiene	
CF5	1	1	1	1	4	
CF9	1	1	1	1	4	
CF2	1	1	1	0	3	
CF10	1	1	1	1	4	
FF8	1	1	1	0	3	
CF7	1	1	1	0	3	
CF3	1	1	1	0	3	
FF5	1	0	1	0	2	
CF6	1	1	1	0	3	
FF3	0	0	0	0	0	
FF4	1	0	1	0	2	
CF8	1	1	1	1	4	
FF2	0	0	1	0	1	
CF4	1	1	1	0	3	
FF1	1	1	1	0	3	
CF1	1	1	1	0	3	
FF7	1	1	1	0	3	
FF9	1	1	1	0	3	
FF6	1	0	1	0	2	
FF10	1	1	1	0	3	
Type	Farm registration	Inspection by CA	Official control			
CF5	1	0	1			
CF9	0	1	1			
CF2	0	0	0			
CF10	1	1	2			
FF8	1	1	2			
CF7	0	1	1			
CF3	0	0	0			
FF5	0	1	1			
CF6	0	1	1			
FF3	1	0	1			
FF4	0	1	1			
CF8	1	1	2			
FF2	1	1	2			
CF4	0	0	0			
FF1	1	1	2			
CF1	0	0	0			
FF7	0	1	1			
FF9	0	1	1			
FF6	0	0	0			
FF10	0	0	0			

## Appendix C. Fit Measures for Structural Equation Modelling (SEM)

lavaan 0.6-19 ended normally after 4 iterations Estimator ML Optimization method NLMINB Number of model parameters 13 Number of observations 20 Model Test User Model: Test statistic 17.832 Degrees of freedom 5 P-value (Chi-square) 0.003 Model Test Baseline Model: Test statistic 143.709 Degrees of freedom 15 P-value 0.000 User Model versus Baseline Model: Comparative Fit Index (CFI) 0.900 Tucker–Lewis Index (TLI) 0.701 Loglikelihood and Information Criteria: Loglikelihood user model (H0) -20.659 Loglikelihood unrestricted model (H1) -11.743 Akaike (AIC) 67.319 Bayesian (BIC) 80.263 Sample-size-adjusted Bayesian (SABIC) 40.187 Root Mean Square Error of Approximation: RMSEA 0.358 90 Percent confidence interval - lower 0.188 90 Percent confidence interval - upper 0.545 P-value H_0: RMSEA <= 0.050 0.004 P-value H_0: RMSEA >= 0.080 0.993 Standardized Root Mean Square Residual: SRMR 0.045 Parameter Estimates: Standard errors Standard Information Expected Information-saturated (h1) model Structured Regressions: Estimate Std.Err z-value P(>|z|) Safety ~ Vdrugcontrol 0.670 0.030 22.347 0.000 HandH 0.395 0.030 13.165 0.000 Vdrugcontrol ~ Business 0.097 0.166 0.584 0.559 Challenges -0.466 0.165 -2.822 0.005 Knowledge 0.531 0.166 3.206 0.001 Official -0.081 0.173 -0.469 0.639 HandH ~ Business -0.038 0.125 -0.305 0.761 Challenges -0.497 0.124 -4.015 0.000 Knowledge 0.617 0.124 4.962 0.000 Official 0.159 0.130 1.228 0.219 Variances: Estimate Std.Err z-value P(>|z|) .Safety 0.012 0.004 3.162 0.002 .Vdrugcontrol 0.489 0.155 3.162 0.002 .HandH 0.276 0.087 3.162 0.002

## References

[1] Nhem S. (1999). Cambodia: Country Context.

[2] Pha S. (2020). Chicken Farming Adds an Income Source and Improves Livelihoods for Villagers.

[3] Joffre O.M., Freed S., Bernhardt J., Teoh S.J., Sambath S., Belton B. (2021). Assessing the Potential for Sustainable Aquaculture Development in Cambodia. Front. Sustain. Food Syst..

[4] Leap H. (2014). National Aquaculture Sector Overview of Cambodia.

[5] National Institute of Statistics (2020). Cambodia Inter-Censal Agriculture Survey 2019 (Cias19)—Final Report.

[6] San S.P., Chea R., Grace D., Roesel K., Tum S., Young S., Charaslertrangsi T., Zand N., Thombathu S.S., Thorng R. (2024). Biological Hazards and Indicators Found in Products of Animal Origin in Cambodia from 2000 to 2022: A Systematic Review. Int. J. Environ. Res. Public Health.

[7] Hyder S., Sievers B.L., Flamand C., TagoPacheco D., Chan M., Claes F., Karlsson E.A. (2023). Influx of Backyard Farming with Limited Biosecurity Due to the COVID-19 Pandemic Carries an Increased Risk of Zoonotic Spillover in Cambodia. Microbiol. Spectr..

[8] Pelić D.L., Radosavljević V., Pelić M., Baloš M.Ž., Puvača N., Jug-Dujaković J., Gavrilović A. (2024). Antibiotic Residues in Cultured Fish: Implications for Food Safety and Regulatory Concerns. Fishes.

[9] Lay K.S., Vuthy Y., Song P., Phol K., Sarthou J.L. (2011). Prevalence, Numbers and Antimicrobial Susceptibilities of Salmonella Serovars and *Campylobacter* spp. in Retail Poultry in Phnom Penh, Cambodia. J. Vet. Med. Sci..

[10] Vuthy Y., Lay K.S., Seiha H., Kerleguer A., Aidara-Kane A. (2017). Antibiotic susceptibility and molecular characterization of resistance genes among Escherichia coli and among Salmonella subsp. in chicken food chains. Asian Pac. J. Trop. Biomed..

[11] Nadimpalli M., Vuthy Y., de Lauzanne A., Fabre L., Criscuolo A., Gouali M., Huynh B.-T., Naas T., Phe T., Borand L. (2019). Meat and Fish as Sources of Extended-Spectrum β-Lactamase–Producing *Escherichia coli*, Cambodia. Emerg. Infect. Dis..

[12] Nadimpalli M., Fabre L., Yith V., Sem N., Gouali M., Delarocque-Astagneau E., Sreng N., Le Hello S., Raheliarivao B.T., The BIRDY Study Group (2019). CTX-M-55-type ESBL-producing *Salmonella enterica* are emerging among retail meats in Phnom Penh, Cambodia. J. Antimicrob. Chemother..

[13] Trongjit S., Angkittitrakul S., Chuanchuen R. (2016). Occurrence and molecular characteristics of antimicrobial resistance of *Escherichia coli* from broilers, pigs and meat products in Thailand and Cambodia provinces. Microbiol. Immunol..

[14] Trongjit S., Angkititrakul S., Tuttle R.E., Poungseree J., Padungtod P., Chuanchuen R. (2017). Prevalence and antimicrobial resistance in *Salmonella enterica* isolated from broiler chickens, pigs and meat products in Thailand–Cambodia border provinces. Microbiol. Immunol..

[15] Rodriguez C., Mith H., Taminiau B., Bouchafa L., Van Broeck J., Soumillion K., Ngyuvula E., García-Fuentes E., Korsak N., Delmée M. (2021). First isolation of Clostridioides difficile from smoked and dried freshwater fish in Cambodia. Food Control.

[16] Kingdom of Cambodia (2022). Law on Food Safety.

[17] Thompson L., Vipham J., Hok L., Ebner P. (2021). Towards improving food safety in Cambodia: Current status and emerging opportunities. Glob. Food Secur..

[18] Mosimann S., Ouk K., Bello N.M., Chhoeun M., Thompson L., Vipham J., Hok L., Ebner P. (2023). Describing food safety perceptions among growers and vendors in Cambodian informal vegetable markets. Front. Sustain. Food Syst..

[19] Fisheries Administration (2023). The National Residue Monitoring Plan for Aquaculture Products.

[20] Rosseel Y. (2012). lavaan: An *R* Package for Structural Equation Modeling. J. Stat. Softw..

[21] The Association of Southeast Asian Nations Ministers on Agriculture and Forestry (2022). Standard on ASEAN Good Aquaculture Practices for Food Fish.

[22] Thongpalad K., Kuwornu J.K.M., Datta A., Chulakasian S., Anal A.K. (2019). On-farm food safety knowledge, attitudes and self-reported practices of layer hen farmers. Br. Food J..

[23] (1969). General Principles of Food Hygiene.

[24] Jimenez C.E.P., Keestra S., Tandon P., Cumming O., Pickering A.J., Moodley A., Chandler C.I.R. (2023). Biosecurity and water, sanitation, and hygiene (WASH) interventions in animal agricultural settings for reducing infection burden, antibiotic use, and antibiotic resistance: A One Health systematic review. Lancet Planet. Health.

[25] Angkor Research (2023). Survey on the Status of Aqua-Medicines, Drugs, and Chemical Use in Cambodian Aquaculture.

[26] Malijan G.M., Howteerakul N., Ali N., Siri S., Kengganpanich M., Nascimento R., Booton R.D., Turner K.M., Cooper B.S., Meeyai A. (2022). A scoping review of antibiotic use practices and drivers of inappropriate antibiotic use in animal farms in WHO Southeast Asia region. One Health.

[27] Coyne L., Benigno C., Giang V.N., Huong L.Q., Kalprividh W., Padungtod P., Patrick I., Ngoc P.T., Rushton J. (2020). Exploring the Socioeconomic Importance of Antimicrobial Use in the Small-Scale Pig Sector in Vietnam. Antibiotics.

[28] Nohrborg S., Nguyen-Thi T., Xuan H.N., Lindahl J., Boqvist S., Järhult J.D., Magnusson U. (2024). Understanding Vietnamese chicken farmers’ knowledge and practices related to antimicrobial resistance using an item response theory approach. Front. Vet. Sci..

[29] Lim J.M., Chhoun P., Tuot S., Om C., Krang S., Ly S., Hsu L.Y., Yi S., Tam C.C. (2021). Public knowledge, attitudes and practices surrounding antibiotic use and resistance in Cambodia. JAC-Antimicrob. Resist..

[30] Ström G., Boqvist S., Albihn A., Fernström L.-L., Andersson Djurfeldt A., Sokerya S., Sothyra T., Magnusson U. (2018). Antimicrobials in small-scale urban pig farming in a lower middle-income country—Arbitrary use and high resistance levels. Antimicrob. Resist. Infect. Control.

[31] Gibson J.S., Wai H., Oo S.S.M.L., Hmwe E.M.M., Wai S.S., Htun L.L., Lim H.P., Latt Z.M., Henning J. (2020). Antimicrobials use and resistance on integrated poultry-fish farming systems in the Ayeyarwady Delta of Myanmar. Sci. Rep..

[32] Shao Y., Wang Y., Yuan Y., Xie Y. (2021). A systematic review on antibiotics misuse in livestock and aquaculture and regulation implications in China. Sci. Total Environ..

[33] Kiambi S., Mwanza R., Sirma A., Czerniak C., Kimani T., Kabali E., Dorado-Garcia A., Eckford S., Price C., Gikonyo S. (2021). Understanding Antimicrobial Use Contexts in the Poultry Sector: Challenges for Small-Scale Layer Farms in Kenya. Antibiotics.

[34] Kemunto N.P., Dang-Xuan S., Luu-Thi-Hai Y., Nguyen-Xuan H., Ibayi E.L., Nielsen S.S., Nguyen-Viet H., Moodley A., Muloi D.M. (2026). Patterns and factors influencing antibiotic use among poultry farmers in Thai Nguyen province, Vietnam. Prev. Vet. Med..

