# Supplementary material for: Food Safety Implementation and Associated Challenges: Insights from Cambodia’s Pangasius Fish and Chicken Farms
Source: Vet Sci. 2026 Apr 15;13(4):380. doi: 10.3390/vetsci13040380 (PMC13119585; doi:10.3390/vetsci13040380)
Supplement: Supplementary file 1 [file vetsci-13-00380-s001.zip › vetsci-4146926-supplementary.pdf]

# Statistical analysis

# Date May 10, 2025

| | |
|---|---|
| Data file | revdsem |
| R script | lavaan |
| Analysis description | |

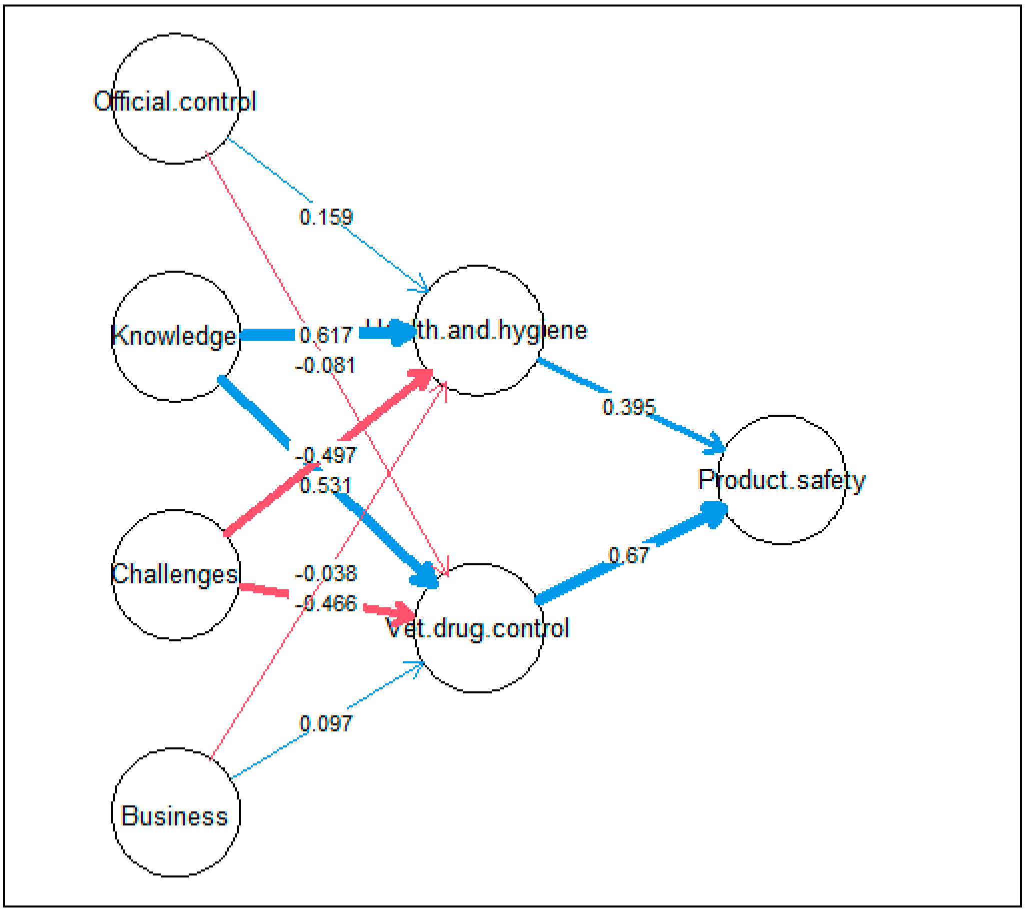


```
Regressions:
                       Estimate  Std.Err  z-value  P-value
  Product.safety ~
    Vet.drug.cntrl        0.670    0.030   22.347    0.000
    Health.nd.hygn        0.395    0.030   13.165    0.000
  Vet.drug.control ~
    Business              0.097    0.166    0.584    0.559
    Challenges           -0.466    0.165   -2.822    0.005
    Knowledge             0.531    0.166    3.206    0.001
```

| | | | | |
|---|---|---|---|---|
| Official.cntrl | -0.081 | 0.173 | -0.469 | 0.639 |
| Health.and.hygiene ~ | | | | |
| Business | -0.038 | 0.125 | -0.305 | 0.761 |
| Challenges | -0.497 | 0.124 | -4.015 | 0.000 |
| Knowledge | 0.617 | 0.124 | 4.962 | 0.000 |
| Official.cntrl | 0.159 | 0.130 | 1.228 | 0.219 |
| | | | | |
| | | | | |
| | | | | |
| | | | | |
| | | | | |
| | | | | |
| | | | | |
| | | | | |
